# The Association Between an Enteral Nutrition Protocol and Achievement of Target Energy Intake in Critically Ill COVID-19 Patients: A Retrospective Observational Study

**DOI:** 10.7759/cureus.105682

**Published:** 2026-03-23

**Authors:** Masayuki Kaku, Satoka Nakagawa, Ayaka Okada, Akiho Yamashita, Satomi Inoue, Hiroshi Ono, Takeshi Nishikawa

**Affiliations:** 1 Graduate School of Medical Science, Kumamoto University, Kumamoto, JPN; 2 Nutrition, NHO Kumamoto Medical Center, Kumamoto, JPN; 3 Infectious Diseases, NHO Kumamoto Medical Center, Kumamoto, JPN; 4 Diabetes and Endocrinology, NHO Kumamoto Medical Center, Kumamoto, JPN

**Keywords:** energy intake, enteral nutrition protocol, infection, intensive care, mechanical ventilation

## Abstract

Background and objective

Enteral nutrition (EN) protocols are commonly used to support energy delivery in critically ill patients. However, to our knowledge, no studies have examined the use of EN protocols in critically ill coronavirus disease 2019 (COVID-19) patients. Therefore, we conducted a single-center, retrospective study to examine whether the use of an EN protocol was associated with achieving the target energy intake of 25 kcal/kg/day within seven days after ICU admission in critically ill COVID-19 patients.

Methods

The retrospective study included critically ill COVID-19 patients between April 1, 2020, and March 31, 2023. The primary outcome was the achievement of the target energy intake, and secondary outcomes were clinical outcomes and the frequency of adverse events according to EN protocol use.

Results

Twenty-five patients (16 males; median age: 65.0 years) were included, and EN was initiated at a median of 44.0 (interquartile range (IQR): 33.0-46.5) hours after ICU admission. The EN protocol was used in 15 patients. The EN protocol was used significantly more frequently among patients who met the target energy intake than among those who did not (*p* = 0.015). A multivariable analysis identified the use of the EN protocol as an independent factor associated with achieving the target energy intake (odds ratio (OR) = 11.00; 95% confidence interval (CI): 1.60-75.50; *p* = 0.015). Moreover, clinical outcomes and adverse events did not significantly differ between patients with and without the EN protocol.

Conclusions

Based on our findings, target energy delivery was associated with the use of an EN protocol in critically ill COVID-19 patients.

## Introduction

The national health insurance system in Japan requires the provision of nutritional therapy based on the guidelines [1] of the Japanese Society of Intensive Care Medicine as therapeutic support for critically ill patients. In 2025, the Society published a revised version of the Japanese Critical Care Nutrition Guideline 2024 (JCCNG2024) [2]. The guideline noted that critically ill Japanese patients do not achieve their energy targets and that the use of an enteral nutrition (EN) protocol is desirable when providing nutritional therapy. Wang et al. have reported that EN provided clinical benefits in terms of achieving energy target and shortening ICU length of stay (LOS) [3]. The use of an EN protocol enables early initiation of EN in critically ill patients [4] and effectively increases energy intake during ICU admission [5]. On the other hand, energy intake did not significantly differ between patients with and without an EN protocol, while vomiting increased in patients receiving an EN protocol [5]. Other studies have found no significant differences in clinical outcomes between patients with and without EN protocols [5,6]. Therefore, there is currently no consensus on the use of EN protocols.

In 2020, medical institutions were required to treat coronavirus disease 2019 (COVID-19) patients who required mechanical ventilation (MV) in the ICU [7]. The American Society for Parenteral and Enteral Nutrition (ASPEN) and the European Society for Clinical Nutrition and Metabolism (ESPEN) have established target energy intakes for critically ill COVID-19 patients based on previous evidence [8,9]. The latest JCCNG2024 states that intentional reductions in energy intake relative to energy expenditure should be avoided during the acute phase of critical illness [2]. Our protocol is aligned with the target energy dosage recommendations of JCCNG2024 [2] and ESPEN [8].

Chapela et al. have reported that 25 kcal/kg/day was the target energy intake in many hospitals and that initiation of EN within 48 hours was associated with achieving this target. Patients at risk of malnutrition based on a subjective global assessment (SGA) were also more likely to achieve this energy intake [10]. However, the association between the use of an EN protocol and the achievement of 25 kcal/kg/day in critically ill COVID-19 patients remains unclear. Furthermore, the association between EN protocol use and clinical outcomes and adverse events remains unknown. Accordingly, this study investigated whether the use of an EN protocol was associated with achieving 25 kcal/kg/day by day seven after ICU admission in critically ill COVID-19 patients. We also examined clinical outcomes and the frequency of adverse events in patients with and without the EN protocol.

## Materials and methods

Study design

This was a single-center retrospective observational study conducted on patients admitted to the ICU in the NHO Kumamoto Medical Center.

Survey period and participants

The survey was conducted between April 1, 2020, and March 31, 2023. Eligible participants were Japanese patients with COVID-19 who were admitted to the ICU, required initiation of MV management, and received EN for more than five days during critical illness. Patients were excluded if they were younger than 18 years of age, were pregnant, were receiving extracorporeal membrane oxygenation, or deviated from the EN protocol procedure. Patients were categorized into a protocol application group and a non-protocol application group based on whether the EN protocol was used. EN was initiated when the vasopressor dose was not increased or had been tapered. In the non-protocol application group, EN was initiated and managed according to the discretion of the attending physicians in routine ICU practice.

Overview of the EN protocol

**Figure 1:**
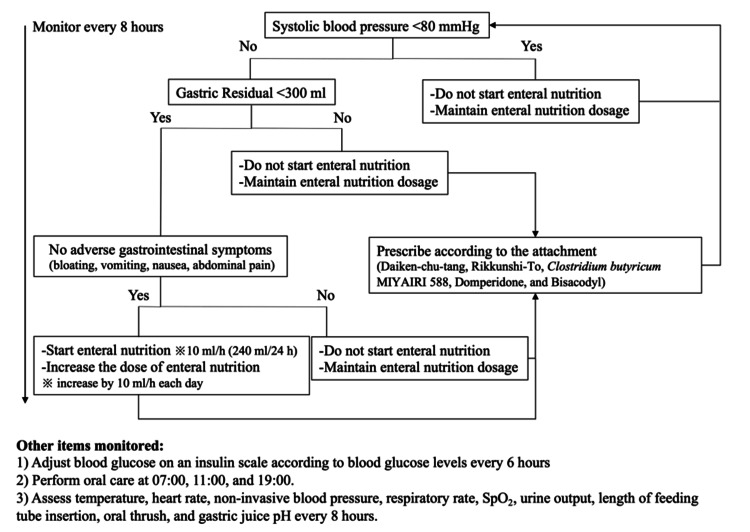
The enteral nutrition protocol

The EN protocol used in the present study was developed with reference to the “Japanese Critical Care Nutritional Guidelines 2016” [1]. The latest JCCNG2024 [2] was published in 2025, and the protocol is consistent with these updated recommendations in terms of criteria for the initiation of EN, administration of enteral formula, management of gastrointestinal complications related to enteral feeding, and use of specific nutritional products [2]. The protocol was initiated within 24-48 hours after ICU admission and consisted of initiation criteria, monitoring items, and stepwise advancement of enteral feeding (Figure 1).

Circulation (Criteria for Initiation and Advancement of EN) 

The EN protocol defined a patient as hemodynamically stable when the systolic blood pressure was ≥ 80 mmHg. If the vasopressor dose was not increased or was tapered off, a decision was made to initiate or increase EN. EN was either discontinued or maintained at the same rate if the vasopressor dose needed to be increased.

Gastrointestinal Symptoms

During the ICU stay, patients were monitored for intestinal peristalsis, gastric residual volume (GRV), and gastrointestinal symptoms (bloating, vomiting, nausea, abdominal pain, diarrhea, and poor bowel movements) at the start of EN, when the EN volume was increased, and every eight hours during EN. If the GRV was < 300 mL per assessment, EN was started or the volume was increased. GRV assessment was mandatory under Japan’s national health insurance system during the study period. In addition, depending on monitoring results, Da-Jian-Zhong-Tang, Rikkunshi-To, *Clostridium butyricum* MIYAIRI 588, domperidone, and bisacodyl were prescribed according to the package inserts.

Blood Glucose (BG)

During the ICU stay, BG was monitored every six hours. If the BG level was ≥ 180 mg/dL, it was controlled using regular insulin or a rapid-acting insulin analogue.

Other Items

Other items monitored were body temperature, heart rate, non-invasive blood pressure, respiratory rate, SpO_2_, urine output, insertion length of the feeding tube, oral thrush, and gastric juice pH. Based on these findings, nutritional therapy was modified or continued as appropriate. These items were monitored at least once every eight hours. Additionally, oral care was performed at 7:00, 11:00, and 19:00.

Suggestions for Nutritional Therapy

The type of EN, administration rate, and dosage were as follows, according to the items monitored: 10 mL/h (240 mL/24 h) of a peptide-based, high protein enteral formula (providing 150 kcal and 9.5 g of protein per 100 mL). Subsequent dosing was increased by 10 mL/h each day; when it was possible to increase the dose to 40 mL/h, a transition from continuous to intermittent feeding was considered with the goal of reaching the target energy intake by day seven after admission to the ICU. At the start of the protocol, a fiber supplement was considered for administration via the side port of the enteral feeding tube; however, it was withheld if the attending physician judged there to be a risk of intestinal obstruction.

Measurements

Age, sex, and BMI, the Sequential Organ Failure Assessment (SOFA) score [11], SGA [12], and the Controlling Nutrition Status (CONUT) score [13] at ICU admission, medical history, the time to EN initiation after ICU admission, whether the EN protocol was used, target energy intake, target protein intake, energy intake during ICU stay, protein intake during ICU stay, whether prone positioning therapy was applied, date of COVID-19 onset, and hematological and biochemical parameters obtained from the first blood sample within 24 hours of ICU admission were collected from medical records. The arterial partial pressure of oxygen to fraction of inspired oxygen (P/F) ratio was also evaluated to assess respiratory function. In addition, the course of BG, vomiting, diarrhea (Bristol stool scale > 5 [14]), GRV, systolic blood pressure, gastrointestinal bleeding, and the duration of MV were followed until death or ICU discharge.

Ideal body weight (IBW) was defined as height (m) × height (m) × 22. The target energy intake was set at 25 kcal/IBW kg/day based on previous studies and clinical guidelines [1,10]. Energy intake was calculated from the time of admission to the ICU to the time of discharge from the ICU and was expressed in kcal/IBW kg/day.

Outcomes

The primary outcome was the association between the use of the EN protocol and the achievement of the target energy intake by day seven after ICU admission. Secondary outcomes included comparisons of clinical outcomes between patients managed with and without the EN protocol, assessment of the frequency of adverse events up to day seven after ICU admission according to protocol use, and evaluation of the frequency of adverse events across subgroups defined by protocol use and achievement of target energy intake.

Statistical analysis

The Strengthening the Reporting of Observational Studies in Epidemiology (STROBE) criteria were followed in reporting the analyses [15]. EZR ver. 1.54 and SPSS ver. 29.0.1 were used for statistical analyses. Categorical variables were presented as numbers (%), and continuous variables were presented as median (interquartile range (IQR)).

To examine the factors associated with achieving the target energy intake as the primary outcome, multivariable logistic regression analysis was performed. Explanatory variables were selected based on clinical relevance and previous studies [10] and included the following variables: the use of the EN protocol, age, sex, the SOFA score at ICU admission, the presence of moderate or severe malnutrition as assessed by SGA at ICU admission, the time to EN initiation after ICU admission, the P/F ratio on ICU admission, the duration of muscle relaxant use, the CONUT score on ICU admission, the presence of prone positioning therapy, BMI ≥ 25 kg/m^2^, the presence of diabetes mellitus, and the presence of chronic renal failure. Multicollinearity among explanatory variables was assessed before the regression analysis, and binary logistic regression with a stepwise (backward elimination) method was performed. Modified Nutrition Risk in the Critically ill (mNUTRIC) scores and SGA were used as nutrition assessment tools in previous studies [10]; however, because the mNUTRIC score was not assessed at this institution, SGA and CONUT scores were used instead.

Regarding the secondary outcomes, Fisher’s exact test, the chi-squared test, or the Mann-Whitney U test (Wilcoxon rank-sum test) was used to compare mortality, the duration of MV, and the ICU LOS between patients with and without the EN protocol. To investigate the frequency of adverse events with and without the EN protocol, the occurrence of BG ≥ 180 mg/dL, BG ≥ 300 mg/dL, vomiting, diarrhea (Bristol stool scale > 5), GRV ≥ 300 mL per assessment, systolic blood pressure < 80 mmHg, and gastrointestinal bleeding were assessed using the chi-squared test and Fisher’s exact test. Furthermore, a p-value < 0.05 was considered statistically significant.

Ethical considerations

This study was conducted in accordance with the principles of the Declaration of Helsinki. The study was approved by the Ethics Review Committee of the NHO Kumamoto Medical Center (approval no: 1263). We employed a retrospective observational design using previously collected medical records, and the requirement for written informed consent was waived. An opt-out consent procedure was applied, whereby information about the study, including its purpose, methods, and data protection, was disclosed on the hospital website. Participants were considered to have provided consent unless they chose to opt out.

## Results

Characteristics

**Figure 2:**
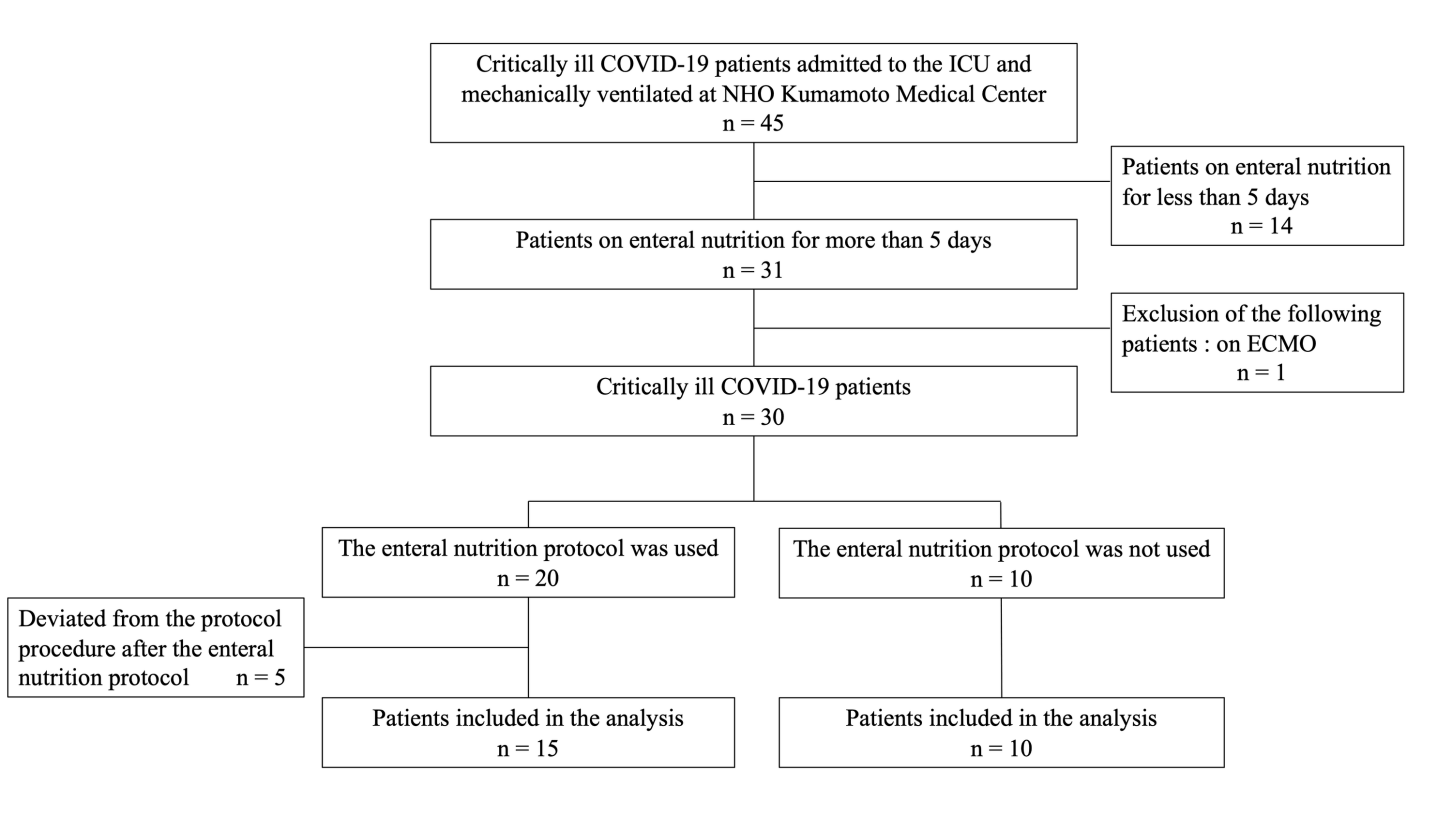
Flowchart depicting the selection of participants COVID-19: coronavirus disease 2019; ECMO: extracorporeal membrane oxygenation; ICU: intensive care unit; NHO: National Hospital Organization

The final analysis included 25 patients (16 males and nine females) with a median age of 65 (IQR: 61-76) years and a BMI of 23.7 (21.1-27.3) kg/m^2^. The median duration from illness onset to MV initiation was seven (6-10) days. EN was initiated 44.0 (33.0-46.5) hours after ICU admission, and the ICU LOS was 14.0 (11.0-17.0) days. EN was initiated within 48 hours in all patients in both groups (44.0 (33.5-46.3) vs. 45.0 (20.6-46.8) hours, *p* = 0.845). At ICU admission, the median SOFA score was 5.0 (3.0-7.0). All patients were classified as malnourished according to the SGA at ICU admission. Prone positioning therapy was used in two patients, and the EN protocol was applied in 15 patients. The number of days from ICU admission to achieving the target energy intake was 6.0 (5.0-7.0) in the achiever group and 10.0 (10.0-10.5) in the non-achiever group. Five non-achievers failed to meet the target energy intake by day seven (Figure 2, Table 1).

**Table 1:** Baseline patient characteristics ^*^P < 0.05. ^**^P < 0.01. ^***^P < 0.001. ^‡^Fisher’s exact test. ^∮^Mann-Whitney U test (Wilcoxon rank-sum test) SGA risk was defined as moderate or severe. ORs (95% CIs) were calculated using Fisher’s exact test, with the non-protocol group as the reference BMI: body mass index; CI: confidence interval; CONUT: Controlling Nutritional Status; IQR: interquartile range; OR: odds ratio; P/F ratio: PaO₂/FiO₂ ratio; SGA: subjective global assessment; SOFA: Sequential Organ Failure Assessment

Variable	All (n = 25)	Protocol application (n = 15)	Non-protocol application (n = 10)	OR (95% CI)	Test statistic	P value	
Age, years, median (IQR)	65.0 (61.0-76.0)	61.0 (58.0-66.5)	73.5 (65.3-81.8)		W = 114.0	0.032	∮^*^
Male sex, n (%)	16 (64.0)	9 (60.0)	7 (70.0)	0.65 (0.08-4.56)	-	0.691	‡
Diabetes (yes), n (%)	11 (44.0)	6 (40.0)	5 (50.0)	0.68 (0.10-4.44)	-	0.697	‡
Chronic kidney disease (yes), n (%)	5 (20.0)	1 (6.7)	4 (40.0)	0.118 (0.002-1.514)	-	0.121	‡
Number of comorbidities, median (IQR)	2 (1-3)	2 (2-3)	2 (1-3)		W = 75.5	1.000	∮
Department (Infectious Disease/Emergency/Nephrology)	15/9/1	15/0/0	0 /9/1	-	χ² = 25.0	<0.001	‡^***^
BMI on admission (kg/m^2^), median (IQR)	23.7 (21.1-27.3)	23.7 (19.8-27.3)	24.2 (22.2-28.5)		W = 90.5	0.405	∮
BMI ≥ 25 kg/m² (yes), n (%)	10 (40.0)	6 (40.0)	4 (40.0)	1.00 (0.15-7.09)	-	1.000	‡
SGA risk on admission (yes), n (%)	25 (100.0)	15 (100.0)	10 (100.0)	-	-	-	‡
CONUT score on admission, median (IQR)	8.0 (7.0-9.0)	8.0 (6.5-9.0)	9.0 (8.3-9.0)		W = 102.5	0.126	∮
The enteral nutrition start time on admission, hours, median (IQR)	44.0 (33.0-46.5)	44.0 (33.5-46.3)	45.0 (20.6-46.8)		W = 71.0	0.845	∮
≤48 hours (yes), n (%)	25 (100.0)	15 (100.0)	10 (100.0)	-	-	-	‡
Target energy intake, kcal/day, median (IQR)	1408 (1304-1570)	1443 (1271-1590)	1356 (1309-1520)		W = 64.0	0.559	∮
Target protein intake, g/day, median (IQR)	73.2 (67.8-81.7)	75.1 (66.1-82.7)	70.5 (68.0-79.1)		W = 64.0	0.559	∮
SOFA score on admission, median (IQR)	5.0 (3.0-7.0)	5.0 (2.5-5.5)	6.5 (3.0-8.8)		W = 102.0	0.137	∮
P/F ratio on admission, median (IQR)	138 (102-172)	124 (101-160)	143 (118-170)		W = 87.0	0.523	∮
Days to severe disease, median (IQR)	7.0 (6.0-10.0)	7.0 (6.0-8.0)	8.5 (5.3-12.0)		W = 84.5	0.614	∮
Administration of muscle relaxants on admission, hours, median (IQR)	21.0 (0.0-42.0)	42.0 (0.0-44.5)	20.0 (3.5-21.0)		W = 48.0	0.134	∮
Prone position therapy (yes), n (%)	2 (8.0)	1 (6.7)	1 (10.0)	0.65 (0.01-55.99)	-	1.000	‡

Factors for achieving target energy delivery

In univariate analysis, the EN protocol was used significantly more frequently in the achiever group than in the non-achiever group (84.6% vs. 33.3%, *p* = 0.015) (Table 2).

**Table 2:** Comparison according to target energy achievement ^*^P < 0.05. ^**^P < 0.01. ^***^P < 0.001. ^‡^Fisher’s exact test. ^∮^Mann-Whitney U test (Wilcoxon rank-sum test). ^a^Excluding five patients who did not achieve the target energy intake. SGA risk was defined as moderate or severe. ORs (95% CIs) were calculated using Fisher’s exact test, with the target non-achievement group as the reference BMI: body mass index; CI: confidence interval; CONUT: Controlling Nutritional Status; IQR: interquartile range; OR: odds ratio; P/F ratio: PaO₂/FiO₂ ratio; SGA: subjective global assessment; SOFA: Sequential Organ Failure Assessment

Variable	Target energy achievement (n = 13)	Target non-achievement (n = 12)	OR (95% CI)	Test statistic	P-value	
Age, years, median (IQR)	62.0 (58.0-66.0)	68.5 (63.3-79.3)		W = 98.5	0.276	∮
Male sex, n (%)	6 (46.2)	10 (83.3)	0.18 (0.01-1.41)	-	0.097	‡
Diabetes (yes), n (%)	6 (46.2)	5 (41.7)	1.19 (0.19-7.72)	-	1.000	‡
Chronic kidney disease (yes), n (%)	2 (15.4)	3 (25.0)	0.56 (0.04-6.04)	-	0.645	‡
Number of comorbidities, median (IQR)	2 (2-3)	2 (1-2)		W = 71.0	0.713	∮
BMI on admission, kg/m^2^, median (IQR)	23.2 (21.1-27.2)	24.3 (21.1-28.5)		W = 91.0	0.496	∮
BMI ≥ 25 kg/m^2^ (yes), n (%)	5 (38.5)	5 (41.7)	0.88 (0.13-5.74)	-	1.000	‡
SGA risk on admission (yes), n (%)	13 (100.0)	12 (100.0)	-	-	-	‡
CONUT score on admission, median (IQR)	8.0 (7.0-9.0)	8.5 (6.8-9.3)		W = 86.5	0.657	∮
Enteral nutrition start time on admission, hours, median (IQR)	39.5 (24.0-46.0)	45.9 (33.0-47.0)		W = 93.5	0.412	∮
≤48 hours (yes), n (%)	13 (100.0)	12 (100.0)	-	-	-	‡
Protocol application (yes), n (%)	11 (84.6)	4 (33.3)	9.78 (1.24-134.32)	-	0.015	‡^*^
Target energy intake, kcal/day, median (IQR)	1339 (1304-1571)	1506 (1330-1562)		W = 89.0	0.567	∮
Target protein intake, g/day, median (IQR)	69.6 (67.8-81.7)	78.4 (69.2-81.2)		W = 89.0	0.567	∮
SOFA score on admission, median (IQR)	5.0 (3.0-6.0)	4.5 (3.0-8.0)		W = 86.5	0.660	∮
P/F ratio on admission, median (IQR)	130 (100-172)	141 (118-157)		W = 86.5	0.663	∮
Days to severe disease, median (IQR)	7.0 (6.0-9.0)	7.5 (5.5-12.0)		W = 85.5	0.701	∮
Administration of muscle relaxants on admission, hours, median (IQR)	34.0 (14.0-42.0)	20.5 (0.0-37.5)		W = 59.0	0.305	∮
Prone position therapy (yes), n (%)	1 (7.7)	1 (8.3)	0.92 (0.01-78.37)	-	1.000	‡
Number of days to achieve the target energy intake after ICU admission^a^, median (IQR)	6.0 (5.0-7.0)	10.0 (10.0-10.5)				-

The following variables were included in the multivariable logistic regression analysis: use of the EN protocol, age, sex, SOFA score at ICU admission, presence of moderate or severe malnutrition as assessed by SGA at admission, time to EN initiation after ICU admission, P/F ratio at ICU admission, duration of muscle relaxant use, CONUT score at ICU admission, prone positioning therapy, BMI ≥25 kg/m^2^, diabetes mellitus, and chronic kidney disease. Multivariable analysis showed that use of the EN protocol was associated with achievement of the target energy delivery (OR: 11.00, 95% CI: 1.60-75.50, *p* = 0.015) (Table 3).

**Table 3:** Factors associated with target energy achievement: logistic regression analysis ^*^P < 0.05. ^‡^Fisher’s exact test. ^∮^Mann-Whitney U test (Wilcoxon rank-sum test) Response variable: target energy achievement. Explanatory variables: age (years), male sex, diabetes, chronic kidney disease, BMI ≥ 25 kg/m², SGA risk, CONUT score, enteral nutrition start time, protocol application, SOFA score, P/F ratio, muscle relaxant use, and prone position therapy. Multivariable analysis was performed after confirming that the variance inflation factor (VIF) was < 10. SGA risk was defined as moderate or severe BMI: body mass index; CI: confidence interval; CONUT: Controlling Nutritional Status; IQR: interquartile range; OR: odds ratio; P/F ratio: PaO₂/FiO₂ ratio; SGA: subjective global assessment; SOFA: Sequential Organ Failure Assessment

Variable	Univariate analysis				Multivariate analysis		
	Target energy achievement (n = 13)	Target non-achievement (n = 12)	P-value		OR (95% CI)	P-value	
Intercept					0.250 (0.053-1.180)	0.080	
Age, years, median (IQR)	62.0 (58.0-66.0)	68.5 (63.3-79.3)	0.276	∮		-	
Male sex, n (%)	6 (46.2)	10 (83.3)	0.097	‡		-	
Diabetes (yes), n (%)	6 (46.2)	5 (41.7)	1.000	‡		-	
Chronic kidney disease (yes), n (%)	2 (15.4)	3 (25.0)	0.645	‡		-	
BMI ≥ 25 kg/m^2^ (yes), n (%)	5 (38.5)	5 (41.7)	1.000	‡		-	
SGA risk on admission (yes), n (%)	13 (100.0)	12 (100.0)	-	‡		-	
CONUT score on admission, median (IQR)	8.0 (7.0-9.0)	8.5 (6.8-9.3)	0.657	∮		-	
Enteral nutrition start time on admission, hours, median (IQR)	39.5 (24.0-46.0)	45.9 (33.0-47.0)	0.412	∮		-	
Protocol application (yes), n (%)	11 (84.6)	4 (33.3)	0.015	‡^*^	11.00 (1.60-75.50)	0.015	^*^
SOFA score on admission, median (IQR)	5.0 (3.0-6.0)	4.5 (3.0-8.0)	0.660	∮		-	
P/F ratio on admission, median (IQR)	130 (100-172)	141 (118-157)	0.663	∮		-	
Administration of muscle relaxants on admission, hours, median (IQR)	34.0 (14.0-42.0)	20.5 (0.0-37.5)	0.305	∮		-	
Prone position therapy (yes), n (%)	1 (7.7)	1 (8.3)	1.000	‡		-	

Comparisons of mortality, duration of MV, and ICU LOS with and without the use of the EN protocol

No significant differences were observed between patients with and without EN protocol use in mortality (1 (6.7%) vs. 1 (10.0%), *p* = 1.000), duration of MV (7.0 (6.0-10.0) vs. 8.5 (7.0-10.5) days, *p* = 0.312), or ICU LOS (14.0 (10.5-15.0) vs. 13.5 (11.3-17.8) days, *p* = 0.656) (Table 4).

**Table 4:** Comparison of clinical outcomes between the protocol and non-protocol groups *P < 0.05. ^‡^Fisher’s exact test; ^∮^Mann-Whitney U test (Wilcoxon rank-sum test) Energy intake ≥ 25 kcal/kg/day was defined as achieving this threshold within seven days after ICU admission. ORs (95% CIs) were calculated using Fisher’s exact test, with the non-protocol application group as the reference CI: confidence interval; ICU: intensive care unit; IQR: interquartile range; LOS: length of stay; MV: mechanical ventilation; OR: odds ratio

Variable	Protocol application (n = 15)	Non-protocol application (n = 10)	OR (95% CI)	Test statistic	P-value	
Energy intake ≥ 25 kcal/kg/day (yes), n (%)	11 (73.3)	2 (20.0)	9.78 (1.24-134.32)	-	0.015	‡^*^
Mortality during ICU stay (yes), n (%)	1 (6.7)	1 (10.0)	0.65 (0.01-55.99)	-	1.000	‡
Duration of MV, days, median (IQR)	7.0 (6.0-10.0)	8.5 (7.0-10.5)		W = 93.5	0.312	∮
ICU LOS, days, median (IQR)	14.0 (10.5-15.0)	13.5 (11.3-17.8)		W = 83.5	0.656	∮

Frequency of adverse events with and without the use of the EN protocol

No significant differences were observed in the frequency of BG ≥ 180 mg/dL, BG ≥ 300 mg/dL, vomiting, diarrhea (Bristol stool scale > 5), GRV ≥ 300 mL per assessment, systolic blood pressure < 80 mmHg, or gastrointestinal bleeding between patients with and without EN protocol use (Table 5). Patients were further stratified into four groups according to EN protocol use and achievement of the target energy intake. No significant differences were observed in adverse event frequencies among these groups (Table 6).

**Table 5:** Comparison of adverse events according to protocol application Data were collected from the initiation of enteral nutrition (ICU admission) through day seven of ICU admission. ORs (95% CIs) were calculated using Fisher’s exact test, with the non-protocol application group as the reference BS: Bristol stool scale; CI: confidence interval; ICU: intensive care unit; OR: odds ratio

Variable	Protocol application (n = 15), n (%)	Non-protocol application (n = 10), n (%)	OR (95% CI)	P-value
Blood glucose ≥ 180 mg/dL (yes)	12 (80.0)	7 (70.0)	1.68 (1.74-16.32)	0.653
Blood glucose ≥ 300 mg/dL (yes)	9 (60.0)	5 (50.0)	1.48 (0.23-9.98)	0.697
Vomiting (yes)	2 (13.3)	1 (10.0)	1.37 (0.06-90.59)	1.000
Diarrhea (BS > 5) (yes)	11 (73.3)	7 (70.0)	1.17 (0.13-9.49)	1.000
Gastric aspirate volume ≥ 300 ml (yes)	0 (0.0)	1 (10.0)	0.0 (0.0-26.0)	0.400
Systolic blood pressure < 80 mmHg (yes)	0 (0.0)	2 (20.0)	0.0 (0.0-3.4)	0.150
Gastrointestinal bleeding (yes)	0 (0.0)	1 (10.0)	0.0 (0.0-26.0)	0.400

**Table 6:** Comparison of adverse events between the four groups Comparisons among the four groups were performed using the χ² test. Patients were classified into four groups according to EN protocol use and energy goal achievement: the non-P/non-E group (EN protocol not used and energy goal not achieved), the non-P/E group (EN protocol not used and energy goal achieved), the P/non-E group (EN protocol used and energy goal not achieved), and the P/E group (EN protocol used and energy goal achieved). BS: Bristol Stool Scale; EN: enteral nutrition; ICU: intensive care unit

Variable	Non-P/non-E (n = 8), (%)	Non-P/E (n = 2), n (%)	P/non-E (n = 4), n (%)	P/E (n = 11), n (%)	Test statistic	P-value
Blood glucose ≥ 180 mg/dL (yes)	5 (62.5)	2 (100.0)	2 (50.0)	10 (90.9)	χ² = 4.25	0.235
Blood glucose ≥ 300 mg/dL (yes)	3 (37.5)	2 (100.0)	1 (25.0)	8 (72.7)	χ² = 5.49	0.139
Vomiting (yes)	1 (12.5)	0 (0.0)	1 (25.0)	1 (9.1)	χ² = 1.00	0.801
Diarrhea (BS > 5) (yes)	6 (75.0)	1 (50.0)	2 (50.0)	9 (81.8)	χ² = 2.00	0.572
Gastric aspirate volume ≥ 300 ml (yes)	1 (12.5)	0 (0.0)	0 (0.0)	0 (0.0)	χ² = 2.21	0.529
Systolic blood pressure < 80 mmHg (yes)	2 (25.0)	0 (0.0)	0 (0.0)	0 (0.0)	χ² = 4.62	0.202
Gastrointestinal bleeding (yes)	1 (12.5)	0 (0.0)	0 (0.0)	0 (0.0)	χ² = 2.21	0.529

## Discussion

The present study investigated whether the use of an EN protocol was associated with achieving an energy intake of 25 kcal/kg/day within seven days after admission to the ICU. The results of both univariate and multivariable analyses showed that the use of the EN protocol was associated with achieving 25 kcal/kg/day. Furthermore, no statistically significant differences were observed in mortality, duration of MV, ICU length of stay, or frequency of adverse events between patients with and without the EN protocol. When EN is administered to patients requiring MV, vomiting and increased GRV must be considered. Furthermore, patients with hemodynamic instability are at risk of intestinal necrosis [16,17], and more time may be needed for the initiation or escalation of EN in patients in the intensive care setting. In a study conducted before the COVID-19 pandemic, the use of an EN protocol was shown to increase energy intake in critically ill patients, while mortality, MV duration, and ICU length of stay were not significantly different [6]. These findings are consistent with the present results.

A study on critically ill COVID-19 patients showed that initiation of EN within 48 hours and risk of malnutrition were associated with achieving 25 kcal/kg/day [10]. However, this study did not examine the use of EN protocols [10]. In the present study, no significant difference was observed in the timing of EN initiation between the group that received the EN protocol and the group that did not. In addition, EN was initiated in all patients within 48 hours of ICU admission. The use of the EN protocol was independently associated with achieving the target energy intake.

The early initiation of EN in the present study, regardless of EN protocol use, may be partly attributed to the widespread adoption of various nutrition therapy guidelines [1,2,18,19] and the Japanese national health insurance system’s active support for early EN initiation. The EN protocol was identified as an independent factor associated with achieving the target energy intake because physicians, nurses, and registered dietitians shared a standardized EN protocol, allowing them to discuss nutritional dosing while monitoring for EN-related complications with a common understanding. This may have facilitated an increase in energy delivery [6]. There is concern that information sharing regarding EN, parenteral nutrition, and stool status may be inconsistent in the management of COVID-19. Therefore, active communication and information sharing between nutrition management staff and bedside staff are important [9]. 

The NUTRIREA-3 trial, a large randomized controlled trial, found no statistically significant differences in 90-day mortality between the 6 kcal/kg/day and 25 kcal/kg/day target groups in the early phase [20]. Moreover, in major clinical trials - including the EAT-ICU trial [21], which aimed at early goal-directed nutrition, as well as the TARGET [22] and PermiT trials [23], which compared energy delivery - no significant differences in clinical outcomes were observed. Initiation of EN within 48 hours is recommended to decrease systemic inflammation and infection due to increased intestinal permeability in critically ill patients and to restore reduced absorptive capacity [1]. We hypothesized that initiation of EN within 48 hours in the present study may be one reason why no statistically significant difference was observed in mortality, MV duration, or ICU LOS.

The present results are consistent with previous findings showing that the use of an EN protocol in critically ill COVID-19 patients may help achieve the target energy delivery and was not associated with an increased frequency of adverse events at the initiation of EN. Therefore, the use of an EN protocol may play an important role in facilitating a rapid increase in energy intake while ensuring safety through ongoing monitoring.

Limitations

Limitations of this study include its single-center, retrospective design and the relatively small number of patients compared with other studies involving critically ill ICU patients. Larger or multicenter studies are needed to draw more generalizable conclusions. In addition, the number of variables included in the multivariable model relative to the sample size may have resulted in statistical instability. Furthermore, there may have been an imbalance in baseline age between the groups. Although age was included as a covariate in the multivariable analysis, residual confounding cannot be completely excluded. Consequently, these findings should be interpreted as associations rather than causal effects.

Furthermore, in previous studies of critically ill COVID-19 patients, illness severity and the prone positioning therapy negatively impacted the achievement of 25 kcal/kg/day [10]. In addition, initiation of EN within 24-48 hours has recently been recommended in Japan in association with medical reimbursement policies. Therefore, because EN may be initiated earlier in our setting, differences in study characteristics between our study and previous studies should be considered. This study provides preliminary exploratory findings regarding the use of an EN protocol in critically ill COVID-19 patients. It also offers insights into the practical implementation of an EN protocol in critically ill COVID-19 patients.

## Conclusions

The use of an EN protocol in critically ill COVID-19 patients was associated with achieving 25 kcal/kg/day without significant differences in clinical outcomes or adverse events. Early initiation of EN combined with a standardized protocol may support energy delivery without a significant increase in adverse events. These suggestive findings provide practical insights into the implementation of protocol-based nutrition management in critically ill patients with COVID-19. Further studies are needed to validate these findings and assess long-term clinical outcomes.
